# Does perfectionism in bipolar disorder pedigrees mediate associations between anxiety/stress and mood symptoms?

**DOI:** 10.1186/s40345-017-0102-8

**Published:** 2017-10-06

**Authors:** Justine Corry, Melissa Green, Gloria Roberts, Janice M. Fullerton, Peter R. Schofield, Philip B. Mitchell

**Affiliations:** 1 School of Psychiatry, University of New South Wales, Randwick, NSW 2031 Australia; 2 Black Dog Institute, Prince of Wales Hospital, Randwick, NSW 2031 Australia; 3 Prince of Wales Hospital, Randwick, NSW 2031 Australia; 4 Neuroscience Research Australia, Randwick, NSW Australia; 5 School of Medical Sciences, University of New South Wales, Randwick, NSW Australia

**Keywords:** Bipolar disorder, Anxiety, Stress, Psychology

## Abstract

**Background:**

Bipolar disorder (BD) and the anxiety disorders are highly comorbid. The present study sought to examine perfectionism and goal attainment values as potential mechanisms of known associations between anxiety, stress and BD symptomatology. Measures of perfectionism and goal attainment values were administered to 269 members of BD pedigrees, alongside measures of anxiety and stress, and BD mood symptoms. Regression analyses were used to determine whether perfectionism and goal attainment values were related to depressive and (hypo)manic symptoms; planned mediation models were then used to test the potential for perfectionism to mediate associations between anxiety/stress and BD symptoms.

**Results:**

Self-oriented perfectionism was associated with chronic depressive symptoms; socially-prescribed perfectionism was associated with chronic (hypo)manic symptoms. Self-oriented perfectionism mediated relationships between anxiety/stress and chronic depressive symptoms even after controlling for chronic hypomanic symptoms. Similarly, socially-prescribed perfectionism mediated associations between anxiety/stress and chronic hypomanic symptoms after controlling for chronic depressive symptoms. Goal attainment beliefs were not uniquely associated with chronic depressive or (hypo)manic symptoms.

**Conclusions:**

Cognitive styles of perfectionism may explain the co-occurrence of anxiety and stress symptoms and BD symptoms. Psychological interventions for anxiety and stress symptoms in BD might therefore address perfectionism in attempt to reduce depression and (hypo)manic symptoms in addition to appropriate pharmacotherapy.

**Electronic supplementary material:**

The online version of this article (doi:10.1186/s40345-017-0102-8) contains supplementary material, which is available to authorized users.

## Background

The high rate of comorbidity between anxiety disorders and bipolar disorder is well established (Pavlova et al. 2015). Anxiety disorders have been shown to precede the onset of bipolar disorder (BD) (de Graaf et al. 2003; Perugi et al. 2001) and there are high rates of anxiety disorders in families affected by BD (Nurnberger et al. 2011; Perich et al. 2015; Merikangas et al. 2014). However, the mechanisms that underlie the comorbidity between anxiety disorders and BD are not fully understood (Mitchell 2015; Provencher et al. 2012). To move beyond purely descriptive studies, we propose that these disorders co-occur because they share common maintaining processes (Harvey et al. 2004). Cognitive styles are important maintaining factors in cognitive models of psychopathology (Harvey et al. 2004), and we propose that the cognitive style of perfectionism may be an explanatory factor in the high co-occurrence of BD and AD’s. Here, we specifically examined perfectionism as a potential mediator of known associations between anxiety and stress symptoms and mood symptomatology (Alloy et al. 2006; Boylan et al. 2004; Corry et al. 2013), in a large family study.

The development of BD is influenced strongly by genetics, accounting for as much as 85% of the variance in who develops mania (McGuffin et al. 2003). In families, a summary of 5 studies estimated that the risk of an affective disorder in first degree relatives of those with BD ranges from 24 to 31% and the risk of BD ranges from 7 to 22% (Merikangas et al. 2002). Given this high genetic contribution to the development of BD, there remains the task of identifying how and when this biological vulnerability is expressed with more proximal biological, psychological and environmental triggers being important factors (Johnson 2005; Jones and Bentall 2008) BD pedigrees have higher rates of Schizoaffective Disorder (BD type), BD-I, BD-II and Major Depressive Disorder that control families (Gershon et al. 1982). There is also emerging evidence that the family environment plays an important role in influencing the development of beliefs and attitudes related to achievement and attainment of goals (Johnson 2005; Chen and Johnson 2012). Hence, studies of extended families with some members diagnosed with BD may be useful to determine the contribution of perfectionistic cognitions and beliefs relating to goal attainment to anxiety, stress, and other core mood symptoms such as depression and hypomania.

Cognitive models of the development of psychopathology propose that cognitive, mood and behavioural symptoms arise when maladaptive beliefs and cognitions are triggered by congruent life events (Beck et al. 1979). Recently, cognitive models of BD symptoms and mood dysregulation have been proposed that attempt to explain both the depressive and (hypo)manic symptoms characteristic of BD (Holmes et al. 2008; Mansell et al. 2007). Mansell et al. (2007) have proposed a transdiagnostic model of mood dysregulation and bipolar disorder, within which it is proposed that self-critical or shaming beliefs may be important in driving anxious thoughts and cognitive appraisals of affect, as well as bodily sensations that ultimately influence the ascent into (hypo)mania or descent into depression (Mansell et al. 2007). In parallel, a separate cognitive model of BD emphasizes the role that anxiety plays in the development of BD symptoms (Holmes et al. 2008). The model of Mansell et al. (2007) in particular draws on findings that perfectionism is a core cognitive style of BD, along with high self-criticism and an emphasis on goal attainment and avoidance of failure (Alloy et al. 2009a; Lam et al. 2004; Mansell and Pedley 2008; Scott et al. 2000). For example, in a prospective study, self-criticism, performance focus and high self-standards interacted with congruent life events to predict both depressive and (hypo)manic symptoms in those with BD-II and cyclothymia (Francis-Raniere et al. 2006). Lam et al. (2004) found that beliefs related to goal attainment distinguished those with BD from normal controls and were correlated with number of hospitalizations for mania and BD episodes in general (Lam et al. 2004). Hewitt et al. (1998) examined perfectionism as a multidimensional construct in a mixed sample of unipolar and BD patients. Hewitt found that *self*-*oriented* perfectionism (whereby the individual has high standards for themselves) was uniquely associated with chronic depression symptoms, while *socially*-*prescribed* perfectionism (whereby individuals perceive that others have high standards for them) was been uniquely associated with chronic (hypo)manic symptoms (Hewitt et al. 1998). A perfectionistic cognitive style has also been associated with anxiety and stress symptoms (Hewitt and Flett 2002; Frost and DiBartolo 2002; Wheeler et al. 2011). Previous associations with perfectionism extend to variations in state and trait anxiety (Flett et al. 1995), social anxiety disorder (Juster et al. 1996), obsessive–compulsive disorder (Antony et al. 1998), panic disorder (Antony et al. 1998) and a tendency to worry (Chang 2000). Consistent with this, greater levels of perfectionism have also been associated with greater cortisol responses to stress in situations designed to induce a fear of negative evaluation (Wirtz et al. 2007) a core cognitive style in social anxiety disorder. These findings, which confirm perfectionism as a key cognitive style in both BD and the anxiety disorders, provided the impetus to examine perfectionism as a potential mediator of known associations between anxiety and stress symptoms and mood symptomatology.

Consistent with the psychological models of BD symptoms (Holmes et al. 2008; Mansell et al. 2007), we have previously reported that anxiety and stress symptoms mediated the effects of self-critical perfectionism and goal attainment beliefs on current bipolar depressive symptoms after controlling for current hypomanic symptoms (Corry et al. 2013). For hypomanic symptoms, stress symptoms were a significant mediator of the relationship between self-critical perfectionism and current hypomanic symptoms; however, these mediation models were no longer significant after controlling current depressive symptoms. Similar findings were reported by O’Garro-Moore et al. (2015) using prospective data from those with bipolar spectrum disorders. Of the six cognitive styles examined as mediators, only perfectionism was a significant mediator of the relationship between the presence of an anxiety disorder and depressive symptoms. No cognitive styles significantly mediated the relationship between anxiety disorder and hypomanic symptoms.

This study thus examined the hypotheses that perfectionism and goal attainment values would mediate the associations between (subclinical) anxiety/stress symptoms and hypomanic and depressive symptomatology, in BD patients and their unaffected relatives. We chose to extend the work of Hewitt et al. by examining perfectionism as a multidimensional construct in a family sample. We hypothesized that: (1) self-oriented perfectionism would mediate the relationship between anxiety and stress symptoms and chronic depressive symptoms; and (2) socially-prescribed perfectionism and goal attainment values would mediate the relationship between stress and anxiety symptoms and chronic (hypo)manic symptoms. In line with Hewitt et al. (1998) findings, these predictions reflected our expectation that: (1) self-oriented perfectionism would be uniquely related to chronic depression depressive symptoms; and (2) socially-prescribed perfectionism and goal attainment values would be uniquely related to chronic (hypo)manic symptoms.

## Methods

### Participants

The participants in the current study had previously been recruited through a BD molecular genetics pedigree research study (McAuley et al. 2009; Fullerton et al. 2010). All families were ascertained after initial consultation with a proband with BD (using the Family Interview for Genetic Studies; FIGS). Each pedigree member provided informed consent prior to inclusion in the study. All individuals were assessed using the Diagnostic Interview for Genetic Studies (DIGS) (Nurnberger et al. 1994), with interviews being undertaken by experienced medical practitioners, psychologists and psychiatric nurses trained in this instrument. Information obtained from the FIGS, DIGS and medical records was used to generate best-estimate Research Diagnostic Criteria (RDC) diagnoses for Bipolar I disorder (BD-I), Bipolar II Disorder (BD-II), Schizoaffective Disorder Manic Type (SZMA) or Recurrent Unipolar Major Depression (RUD). As we were interested in examining both sub-syndromal and syndromal mood disorder symptoms, we also included those participants who did not meet formal RDC criteria for a mood disorder but who may still be at greater risk of developing symptomatology due to increased familial risk.

The questionnaire for the current study was posted to all contactable first and second degree relatives of the proband in the 67 pedigrees (*n* = 735) with a return deadline of 24 days. After 37 days, 267 responses were received. At 45 days, a secondary mail-out was conducted to those individuals who had failed to respond, which yielded an additional 76 responses giving a 46.6% response rate. Of the 343 individuals who completed the questionnaires, 325 individuals responded to over 95% of the 164 items, and 297 individuals responded to over 99% of items. Of the 67 families included in the mail-out, 47 had 2 or more members complete over 95% of the survey. Missing data was imputed using the mean response of other items within the same questionnaire or sub-scale as applicable (Tabachnick and Fidell 2013). Respondents completing less than 95% of the survey were not included in the analysis in order to reduce the impact of non-randomly missing datapoints in the dataset (Tabachnick and Fidell 2013).

### Measures

#### Internal State Scale (ISS)

The Internal State Scale is a 15-item self-report measure designed to provide a simple mood state ascertainment for those with BD (Bauer et al. 1991). There are four sub-scales—Activation, Well Being, Depression Index and Perceived Conflict—which are rated using a visual analogue line scale. An algorithm using scores from the Activation and Wellbeing sub-scales identifies individuals in euthymic, depressed, manic and mixed states. The ISS has been found to discriminate well, with moderate kappas found between clinician-determined diagnostic status and that determined by the ISS (Bauer et al. 1991; Bauer et al. 2000; Glick et al. 2003).

#### General Behaviour Inventory (GBI)

The GBI is a measure of chronic-intermittent forms of affective disorders and identifies the full range of severity of unipolar and BD—from sub-syndromal to syndromal (Depue et al. 1981, 1989). It consists of three clusters of items: depressive, hypomanic and biphasic (defined as the tendency to vacillate between depression and hypomania in the same item). The GBI has 73 items that are scored on a 0–4 Likert scale related to the chronicity of the symptom described. It is possible to use the scoring system of the GBI as a Likert scale yielding continuous scores of affective disturbance or dysregulation in the domains of depressive, hypomanic and biphasic symptoms. We used a selection of ten items from the depression domain of which the majority selected being consistent with a recent factor analysis informing the development of a shorter version of the GBI (Youngstrom et al. 2013). The items were: (1) Have you had periods of sadness and depression when almost everything gets on your nerves and makes you irritable or angry (other than related to the menstrual cycle)?; (2) Have there been times of several days or more when you were so sad that it was quite painful or you felt that you couldn’t stand it?; (3) Have there been times when you looked back over your life and could see only failures or hardships?; (4) Have there been times when you were feeling low and depressed, and you also had to struggle very hard to control inner feelings of rage or an urge to smash or destroy things?; (5) Have there been times when you exploded at others and afterwards felt bad about yourself?; (6) Have there been times when you hated yourself or felt that you were stupid, ugly, unlovable, or useless? (7) Have there been times of several days or more when you really got down on yourself and felt worthless?; (8) Have you had periods when it seemed that the future was hopeless and things could not improve?; (9) Have there been periods lasting several days or more when you were so down in the dumps that you thought you might never snap out of it?; and (10) Have there been times when you have felt that you would be better off dead? All 27 items for the hypomanic and biphasic domains were administered. The GBI has been extensively validated and possesses good psychometric properties (Depue et al. 1981, 1989).

#### Depression, Anxiety and Stress Scale (DASS)

The DASS is a 42-item scale measuring the negative emotional states of depression, anxiety and stress with each sub-scale having 14 items (Lovibond and Lovibond 1995a). In the current study, we focused on the Anxiety and Stress sub-scales. The anxiety sub-scale is a measure of the acute autonomic fear response. The stress sub-scale measures the state of persistent arousal and tension with a low threshold for becoming upset or frustrated. For clinical utility, DASS scores can be categorized into normal, mild, moderate, severe and extreme severe categories for each emotional state (Lovibond and Lovibond 1995a). The DASS has been shown to have good psychometric properties (Lovibond and Lovibond 1995a; Lovibond and Lovibond 1995b).

#### Multidimensional Perfectionism Scale (MPS)

The MPS is a 45-item scale assessing three dimensions of perfectionism: (1) self-oriented perfectionism (i.e. the tendency to establish perfectionistic standards for oneself); (2) socially-prescribed perfectionism (i.e. The tendency to believe that others have unrealistic standards for oneself); and (3) other-oriented perfectionism (i.e. the tendency to have perfectionistic expectations of others) (Hewitt and Flett 1991). The MPS has been shown to have good reliability, internal consistency and validity (Hewitt and Flett 1991; Hewitt et al. 1991). The present study focused on self-oriented perfectionism (SO) and socially-prescribed perfectionism (SP) and therefore only these sub-scales of the MPS were administered.

#### Dysfunctional Attitudes Scale (DAS)

The original DAS consists of two 40-item parallel questionnaires items that tap various cognitive schemas proposed to place individuals at risk of depression (Weissman and Beck 1978). Factor analytic investigation of this scale has yielded various factors that relate to these cognitive schemas. We used the goal attainment factor from Lam et al. (2004) which is proposed to measure the intensity of beliefs related to positive affect, extraversion and achievement-striving (Lam et al. 2004). The goal attainment factor was found to be highly reliable (6 items; *α* = 0.79).

### Statistical analyses

Analyses were conducted using SPSS Statistics package and MPlus using procedures to account for the nested nature of the dataset. Pearson correlations were calculated for bivariate associations between the measures of cognitive style (perfectionism and goal attainment), chronic BD symptomatology (i.e., depression and hypomania), anxiety and stress symptoms, demographics and current episode. In order to test whether perfectionism and goal attainment values were uniquely related to BD symptomatology, we used generalized estimating equations (GEE procedure) to generate two regression models: one for chronic depressive symptoms and one for chronic (hypo)manic symptoms. We controlled for the effects of gender, age, presence vs absence of current BD episode and chronic depressive or (hypo)manic symptoms (depending on the independent variable). All variables were entered in one block so that the beta for any one independent variable represents the degree of association between that dependent variable and the independent variable after controlling for all other dependant variables in the model (Tabachnick and Fidell 2013).

Mediation analyses were conducted using nonparametric bootstrapping methods to test the significance of the proposed mediators in our models using Mplus (Muthén et al. 1998). Two separate mediation models were used for both chronic depressive symptoms and chronic (hypo)manic symptoms with the analysis comprising of four mediation models in total. In the first two models, the tendency to experience chronic depressive symptoms was the dependent variable, self-oriented and socially-prescribed perfectionism were entered simultaneously as the potential mediators, and anxiety and stress symptoms were the independent variables. These analyses were repeated for (hypo)manic symptoms as the dependent variable.

For demonstration of mediation, the independent variable (anxiety and stress symptoms) must be related to the dependent variable (chronic depressive and hypo/manic symptoms), the independent variable must be related to the proposed mediating variables (self-oriented perfectionism and socially-prescribed perfectionism and goal attainment values), and the relationship between the mediating variables and the dependent variable (the indirect effect) should be stronger than the relationship between the independent variable and the dependent variable (the direct effect). Using a nonparametric bootstrapping approach to mediation, the indirect effect is generated as a point estimate with 95% confidence intervals. If zero is not included in the 95% confidence intervals, it can be concluded that the indirect effects are significantly different from zero at *p* < 0.05 (two-tailed) and that partial or full mediation is present. We also included age, gender, the presence of a depressive, (hypo)manic or mixed episode as covariates in our analyses, and the opposing mood state (chronic depressive symptoms or chronic hypomanic/biphasic symptoms).

We also conducted additional analyses examining these four models separately for the affected group and unaffected group. These are reported in the Additional file 1.

## Results

The sample comprised 269 subjects from 56 pedigrees of whom 17.5% (*n* = 47) had BD-I; 6.3% (*n* = 17) BD-II; 8.9% (*n* = 24) recurrent unipolar depression; 5.2% (*n* = 14) schizoaffective disorder (bipolar type); and 62.1% (*n* = 167) unaffected relatives who did not meet formal diagnostic criteria for any mood disorder. Descriptive statistics for the total sample are detailed in Table 1. *T* tests between affected and unaffected subjects showed significant differences between the two groups on the DASS stress sub-scale (*p* < 0.001). The same pattern of results was observed for anxiety scores: those with a mood disorder diagnosis had higher anxiety scores (*p* < 0.001). The mean scores for the stress and anxiety sub-scales of the DASS were low—in the normal range according the DASS severity categories (Lovibond and Lovibond 1995b). There were no significant differences between those affected and unaffected on current rates of depressive, (hypo)manic or mixed episodes (as measured by the ISS). Those in the affected groups, however, had higher levels of self-orientated perfectionism (mean = 14.36, SD = 6.12 v. mean = 16.89, SD = 6.86; *t*(267) = −3.15, *p* < 0.001). There were no significant differences between the two groups on measures socially-prescribed perfectionism or goal attainment values.

**Table 1:** Participant characteristics

	Mean (SD)		
	Unaffected (*n* = 167)	Affected (*n* = 102)	Total sample (*n* = 269)
Demographics			
Age, years	51.86 (14.34)	52.51 (14.73)	52.11 (14.46)
Female, % (*n*)	60.5 (101)	60.8 (62)	60.6 (163)
Current episode			
ISS depressive episode, % (*n*)	13.8 (23)	22.5 (46)	17.1 (69)
ISS hypomanic episode, % (*n*)	17.4 (29)	23.5 (24)	19.7 (53)
ISS mixed episodes, % (*n*)	3.0 (5)	6.9 (7)	4.5 (12)
Chronic symptomatology			
GBI depression	13.05 (4.75)	18.71 (6.54)***	15.19 (6.13)
GBI hypomanic/biphasic	33.41 (10.21)	44.71 (14.12)***	37.70 (13.03)
DASS anxiety	3.68 (3.53)	7.17 (4.27)***	5.00 (4.18)
DASS stress	4.13 (3.42)	7.04 (4.37)***	5.23 (4.05)
Cognitive styles			
MPS: self-orientated perfectionism	14.36 (6.12)	16.89 (6.86)***	15.31 (6.51)
MPS: socially-prescribed perfectionism	21.70 (6.43)	21.96 (7.06)	21.80 (6.67)
DAS: goal attainment values	24.23 (7.35)	23.36 (8.18)	23.94 (7.67)

*ISS* Internal State Scale, *GBI Depression* General Behaviour Inventory Depression sub-scale, *GBI Hypomanic/Biphasic* General Behaviour Inventory Biphasic/Hypomanic sub-scale, *DASS Anxiety* Depression Anxiety Stress Scale-Anxiety sub-scale, *DASS Stress* Depression Anxiety Stress Scale-Stress scale, *MPS* Multidimensional Perfectionism Scale, *DAS* Dysfunctional Attitudes Scale

*** *p* < 0.001

### Correlation analyses

Table 2 shows Pearson’s *r* correlations between the measures included in the analysis. All variables were correlated in the expected directions. Both measures of perfectionism (self-oriented and socially-prescribed) and goal attainment values were significantly correlated with chronic depression and (hypo)manic symptoms. Anxiety and stress symptoms were also significantly correlated with chronic depression and (hypo)manic symptoms.

**Table 2:** Zero-order correlations for all variables

	1	2	3	4	5	6	7	8	9	10	11
Age	–										
Gender	−0.06	–									
ISS depressive episode	0.08	0.00	–								
ISS hypomanic episode	−0.09	−0.08	−0.23	–							
ISS mixed episode	−0.01	−0.05	−0.10	−0.11	–						
GBI depression	−0.14*	0.05	0.15*	0.23**	0.15*	–					
GBI hypomanic/biphasic	−0.13*	−0.13*	0.06	0.34**	0.17**	0.73**	–				
DASS anxiety	−0.15*	−0.08	0.14*	0.32**	0.20**	0.74**	0.71**	–			
DASS stress	−0.21**	0.08	0.15*	0.33**	0.17**	0.74**	0.72**	0.85**	–		
MPS self-oriented	−0.09	−0.13*	0.10	0.24**	0.10	0.48**	0.45**	0.45**	0.47**	–	
MPS socially-prescribed	−0.18**	−0.10	−0.10	0.18**	0.12*	0.31**	0.40**	0.31**	0.32**	0.54**	–
DAS goal attainment	−0.16**	−0.14*	−0.06	0.25**	0.07	0.29**	0.38**	0.31**	0.34**	0.49**	0.55**

*ISS* Internal State Scale, *GBI depression* General Behaviour Inventory Depression sub-scale, *GBI hypomanic/biphasic* General Behaviour Inventory Biphasic/Hypomanic sub-scale, *DASS anxiety* Depression Anxiety Stress Scale-Anxiety sub-scale, *DASS stress* Depression Anxiety Stress Scale-Stress scale, *MPS self-oriented* Multidimensional Perfectionism scale Self-Oriented sub-scale, *MPS socially-prescribed* Multidimensional Perfectionism scale Socially-Prescribed sub-scale, *DAS goal attainment* Dysfunctional Attitudes Goal Attainment Factor

* *p* < 0.05; ** *p* < 0.01 (these analyses are exploratory and therefore no adjustments for multiple comparisons have been made)

#### Generalized estimating equation regression models

The first model demonstrates that self-oriented perfectionism was the only significant predictor of chronic depressive symptoms (as measured by the GBI), after controlling for other independent variables (Table 3). No other cognitive style predicted depressive symptomatology, but other significant predictors were a current depressive episode (as determined by the ISS) and chronic (hypo)manic symptomatology (as measured by the GBI). Being female was also associated with higher levels of chronic depressive symptoms.

**Table 3:** Generalized Estimating Equations Using Multidimensional Perfectionism Scale Dimensions and Goal Attainment Values to Predict Chronic Depressive and Chronic Hypomanic/Biphasic symptoms

Variable	Beta	SE
Predicting chronic unipolar symptoms		
Age	−0.02	0.02
Gender	−0.19	0.47***
ISS: depressed	−1.42	0.63*
ISS: hypomanic	0.10	0.82
ISS: mixed	−1.04	1.57
GBI: hypomanic/biphasic	0.31	0.02***
DAS: goal attainment factor	−0.02	0.04
MPS: self-oriented perfectionism	0.21	0.06***
MPS: socially-prescribed perfectionism	−0.04	0.05
Predicting chronic hypomanic/biphasic symptoms		
Age	−0.002	0.03
Gender	3.25	1.05**
ISS: depressed	−1.04	0.50
ISS: hypomanic	−5.41	1.45***
ISS: mixed	−4.46	2.49^#^
GBI: depression	1.34	0.13***
DAS: goal attainment factor	0.14	0.07^#^
MPS: self-oriented perfectionism	−0.04	0.13
MPS: socially-prescribed perfectionism	0.23	0.09**

*ISS* Internal State Scale, *GBI* General Behaviour Inventory, *DAS* Dysfunctional Attitudes Scale, *MPS* MultiDimensional Perfectionism Scale

^#^ *p* < 0.10, * *p* < 0.05, ** *p* < 0.01, *** *p* < 0.001

The second model showed that only socially-prescribed perfectionism significantly predicted chronic (hypo)manic symptomatology, after controlling for the other independent variables (Table 3). Self-oriented perfectionism was not a significant predictor. Other significant predictors of chronic (hypo)manic symptomatology were chronic depressive symptoms and a current hypomanic episode (as determined by the ISS). Goal attainment values only approached significance in this model (*p* < 0.10). Being male was also associated with higher levels of chronic (hypo)manic symptoms.

#### Mediation analyses

As goal attainment values was not a significant predictor in the generalized estimating equation regression models this cognitive style is not considered further in the mediation models. In the two mediation models where chronic depressive symptoms was the dependent variable, only self-oriented perfectionism mediated the relationship between both anxiety and stress symptoms and chronic depressive symptoms (Table 4). Socially-prescribed perfectionism was not a significant mediator. For anxiety symptoms, the true indirect effects was estimated to lie between 0.05 and 0.17. For stress symptoms, the true indirect effects were estimate to lie between 0.04 and 0.17.

**Table 4:** Summary of mediation results for chronic depressive and hypomanic symptoms with self-oriented perfectionism and socially-prescribed perfectionism as mediators controlling for age, gender and opposing mood state

IV	Effect of IV on DV	Mediators	Effect of IV on M	Effect of M on DV	Indirect effect
Chronic depressive symptoms					
Anxiety	0.60***	SOP	0.70***	0.15**	0.11^a^
		SPP	0.50***	−0.05	−0.02
Stress	0.62***	SOP	0.75***	0.11**	0.12^a^
		SPP	0.53***	−0.02	0.01
Chronic (Hypo)manic symptoms					
Anxiety	0.91***	SOP	0.70***	−0.03	−0.02
		SPP	0.50***	0.30**	0.15^a^
Stress	1.03***	SOP	0.75***	−0.05	−0.04
		SPP	0.53***	0.30**	0.16^a^

*IV* independent variable, *M* mediating variable, *DV* dependent variable, *SOP* self-oriented perfectionism, *SPP* socially-prescribed perfectionism

* *p* < 0.05; ** *p* < 0.01; *** *p* < 0.001

^a^Significant point estimate *p* < 0.05

In the two mediation models where chronic hypomanic symptoms were the dependent variables, socially-prescribed perfectionism mediated the relationship between both anxiety and stress symptoms and chronic hypomanic symptoms. For anxiety symptoms, the true indirect effects were estimated to lie between 0.07 and 0.26. For stress symptoms, the true indirect effects were estimated to lie between 0.08 and 0.27. Self-oriented perfectionism was not a significant mediator.

Separate analyses for probands and non-affected family members are available in the supplementary analysis (see Additional file 1: Tables S1, S2). As the results replicate the combined sample, they were not included in the main results of this paper. The results in both groups are consistent with the combined sample with only self-oriented perfectionism significantly mediating the relationship between anxiety and stress symptoms and chronic depressive symptoms. For hypomanic symptoms, only socially-prescribed perfectionism significantly mediated the relationship between anxiety and stress symptoms and chronic hypomanic symptoms.

## Discussion

The results highlight the potential role of perfectionism as a mechanism explaining the co-occurrence between BD and anxiety. The results further suggest that different dimensions of perfectionism are uniquely associated with chronic depressive and (hypo)manic symptoms. Univariate associations between self- and socially-prescribed perfectionism and BD symptomatology were consistent with previous reports (Lam et al. 2004; Hewitt et al. 1998). Similarly, we confirmed significant relationships between both self- and socially-prescribed perfectionism and anxiety and stress symptoms (Wheeler et al. 2011). Like Hewitt (Hewitt et al. 1998), we found that self-oriented perfectionism was uniquely associated with chronic depressive symptoms after controlling for chronic (hypo)manic symptoms; this suggests a preoccupation with high standards for oneself and a punitive response when mistakes are made, or when perfectionistic standards are not met. Previous studies have found self-oriented perfectionism to be associated with unipolar depressive symptoms (Hewitt and Flett 1993; Hewitt et al. 1996) and there are similarities between the cognitive styles of unipolar and bipolar depression (Scott and Pope 2003). The particular relevance of self-oriented perfectionism for depression (versus socially-prescribed perfectionism) is consistent with research highlighting the role of ruminative, self-focused attention in the onset and maintenance of depression (Nolen-Hoeksema 1991, 2000; Kuehner and Weber 1999). Self-analytical self-focus has been found to influence global negative self-judgments (e.g. “I am a failure”) which increases depressive symptoms (Rimes and Watkins 2005).

For hypomanic symptoms, our findings suggest that an interpersonal focus is more important given that socially-prescribed perfectionism is defined as perceiving that others hold excessively high standards for oneself. We found that socially-prescribed perfectionism was uniquely associated with chronic (hypo)manic symptoms after controlling for chronic depressive symptoms. This is consistent with Shepero et al’s (Shapero et al. 2015) finding that higher public self-consciousness (reflecting a person’s awareness of other’s view of themselves) distinguished BD patients from those with MDD (along with higher rumination on positive affect) (Shapero et al. 2015). Socially-prescribed perfectionism is also considered to be more maladaptive than self-oriented perfectionism, with self-criticism being the most problematic component (Blankstein and Dunkley 2002). In a sample of individuals with BD-II and cyclothymia, Alloy et al. found that higher autonomy (a construct related to perfectionism) and self-criticism predicted a greater likelihood of (hypo)manic episodes over 3.2 year follow-up (Alloy et al. 2009b). Our findings strengthen the hypothesis that a cognitive style characterized by a concern about the negative perceptions of others is associated with hypomanic symptoms.

The second aim of our study was to examine whether perfectionism mediates the relationship between stress and anxiety symptoms and BD symptoms in an attempt to understand mechanisms underlying the high comorbidity between anxiety symptoms and BD. For chronic bipolar depressive symptoms, the mediation models were significant with self-orientated perfectionism found to significantly mediate the relationship between anxiety and stress symptoms and bipolar depressive symptoms. These findings remained after controlling for the presence of chronic (hypo)manic symptoms. These findings are consistent with our previous finding in a clinical sample (Corry et al. 2013). Our hypothesis that socially-prescribed perfectionism and goal attainment values would mediate the relationship between anxiety and stress symptoms and chronic hypomanic symptoms was partially supported. Only socially-prescribed perfectionism (not goal attainment values) was uniquely associated with chronic hypomanic symptoms in the linear regression models in the current study; therefore, we considered only socially-prescribed perfectionism in the mediation models. In mediation models, socially-prescribed perfectionism significantly mediated associations between both anxiety and stress, and chronic hypomanic symptoms after controlling for the presence of chronic depressive symptoms. This finding is inconsistent with our previous study and likely represents methodological differences in the measurement of (hypo)mania (chronic in the present study versus current in our previous study) (Corry et al. 2013). It is important to note that the present findings do not imply that perfectionism is the only mediator between anxiety and stress symptoms and bipolar disorder symptoms. Perfectionism is only a partial mediator in the mediation models, leaving room for other constructs to play a role. For example, the perfectionism literature also highlights the importance of coping styles, emotional regulation strategies and congruent life events to explain the relationship between perfectionism and psychopathology [e.g. (Dunkley and Blankstein 2000; Dunkley et al. 2003)].

Our findings are consistent with current cognitive, transdiagnostic models of BD. Specifically, that self-critical, perfectionistic beliefs are related to both depressive and (hypo)manic symptoms (see Mansell et al. 2007; Mansell 2007) and that anxiety is likely a precursor to depressive symptoms in BD (Holmes et al. 2008). Consistent with Mansell et al.’s and Holmes’ model it is proposed that depressive and/or (hypo)manic symptoms arise from the use of unhelpful emotional regulation strategies (ascent and descent behaviours) in response to anxiety and stress symptoms driven by perfectionistic and/or goal attainment beliefs [see (Mansell 2007) for an illustrative case formulation], (Holmes et al. 2008; Mansell et al. 2007). A key component of Mansell’s model, the conflicted appraisals of an individual’s internal state, was not examined here, but would be crucial in further understanding the transition from stress and anxiety symptoms to depressive and (hypo)manic symptoms. The role that perfectionism may play in these appraisals also warrants further investigation. It may be that anxiety/stress and depression are not merely co-occurring emotional states (or comorbid disorders) but that there is a dynamic, iterative process that occurs within an individual that sometimes produces a predominantly anxious presentation (i.e. meeting criteria for an anxiety disorder) and at other times produces symptoms consistent with a formal mood episode. Indeed, (Mansell 2007) proposes that the manner in which mood fluctuations express themselves over time should determine the diagnosis and that the nature of current symptoms will be a complex result of: (1) experiencing previous vicious cycles; (2) the nature of the underlying beliefs; and (3) the current environment.

The presence of these significant relationships in the current sample, comprising both affected and unaffected relatives within affected families, suggests that these processes are operating outside of formal episodes and may represent intermediate phenotypes in those at genetic risk of developing either depressive or (hypo)manic episodes. Perfectionism has been proposed to have developmental origins (Flett et al. 2002), with maternal criticism when standards are not met (Bleys et al. 2016; Greblo and Bratko 2014) and parental psychological control found to be particularly important (Soenens et al. 2005). Our findings are also consistent with perfectionism being a transdiagnostic psychological risk factor for psychopathology generally (Bieling et al. 2004; Egan et al. 2011). In BD, previous research suggests that family socialization plays a role in shaping beliefs towards goal attainment and achievement that conveys a vulnerability to developing the cognitions that are characteristic of (hypo)mania (Chen and Johnson 2012). Given that the presence of perfectionistic cognitions and anxiety/stress symptoms may be necessary but not the whole picture of risk for experiencing BD symptoms, future research is necessary to better understand what additional risk factors are present that give rise to BD symptomatology against a background of familial risk.

A number of limitations in this study are important to acknowledge. Firstly, the cross-sectional nature of the study means that caution must be exercised when inferring causality. Ultimately longitudinal data will be required to more definitively investigate relationships between perfectionism, anxiety/stress, and mood symptom development over time. The cross-sectional nature of the study also makes it impossible to be certain about the directionality of the relationship between the variables of interest. However, our hypotheses are proposed on the basis of other studies that have used longitudinal designs and have attained similar results (O’Garro-Moore et al. 2015; Alloy et al. 2009b). Secondly, the present study examined anxiety (and stress) symptoms dimensionally rather than using formal categorical diagnostic criteria; to better understand issues of comorbidity with BD, it will be useful to examine these relationships among people with specific categorical anxiety diagnoses, such as those defined by DSM-5. Thirdly, anxiety has been found to influence the course of bipolar disorder in other ways. Higher likelihood of relapse (Otto et al. 2006) increased suicidal behaviour (Dilsaver et al. 2006; Young et al. 1993) and greater number of episodes (Bauer et al. 2005) have all been found to be increased in those with comorbid BD and anxiety disorders compared to those with BD only. While beyond the scope of the current study it would be useful to examine the role of perfectionism mediating the relationship between anxiety and other markers of BD severity. For example, people with BD who exhibit a cognitive style characterized by rigidity and pessimism are less likely to recover from their BD depressive episode or, if able to recover, take longer to do so (Stange et al. 2013). Additionally, we did not measure the occurrence of life events in the current study. The cognitive-diathesis stress model of psychopathology proposes that negative cognitive styles or schemas interact with life events to trigger symptoms of psychopathology (Beck 1967, 1976); specific life events associated with achievement may be particularly relevant to the development of mood episodes in BD (Alloy et al. 2005; Koenders et al. 2014). Lastly, the variables of interest were assessed using retrospective, self-report measures and it therefore it could be argued that responses are unduly influenced by the current mood state of the participants. Future research using experience-sampling methodology would enable the relationships between anxiety and stress symptoms, perfectionism and BD symptoms to be more clearly elucidated.

## Conclusions

In sum, this study provides support for the role of perfectionism in the co-occurrence of anxiety, stress, depression and (hypo)manic symptoms in BD. There are promising treatments for clinical perfectionism (Egan et al. 2014) and also transdiagnostic approaches to targeting both anxiety and BD symptoms (Ellard et al. 2012) that may be useful in psychological treatment approaches. These findings further suggest that BD patients and/or those considered at risk of BD should be routinely screened for the presence of maladaptive perfectionism and considered for the need for psychological intervention to address anxiety, depressive and (hypo)manic symptoms, in addition to appropriate pharmacotherapy.

## Additional file

**Additional file 1.** Supplementary separate analyses for probands and non-affected family members.
