# Supplementary material for: Does perfectionism in bipolar disorder pedigrees mediate associations between anxiety/stress and mood symptoms?
Source: Int J Bipolar Disord. 2017 Oct 6;5:34. doi: 10.1186/s40345-017-0102-8 (PMC5629191; doi:10.1186/s40345-017-0102-8)
Supplement: Supplementary file 1 — Additional file 1. Supplementary separate analyses for probands and non-affected family members. [file 40345_2017_102_MOESM1_ESM.docx]

Supplementary Analyses

*Statistical Analyses*

Mediation analyses were conducted using nonparametric bootstrapping methods in MPlus that accounted for the nested nature of the dataset. For both groups (probands and unaffected relatives) two mediation models were constructed separately for chronic depressive symptoms and chronic hypomanic/biphasic symptoms.

RESULTS

*Mediation analyses for unaffected relatives*

Supplementary Table 1 details the results of the mediation models for the unaffected relatives in the sample. In the two mediation models where ‘chronic depressive symptoms’ was the dependent variable, only self-oriented perfectionism mediated the relationship between both anxiety and stress symptoms and chronic depressive symptoms. Socially-prescribed perfectionism was not a significant mediator. For anxiety symptoms the true indirect effects was estimated to lie between 0.04 and 0.20. For stress symptoms the true indirect effects were estimate to lie between 0.04 and 0.19.

In the two mediation models where ‘chronic (hypo)manic symptoms’ was the dependent variable, only socially-prescribed perfectionism mediated the relationship between both anxiety and stress symptoms and chronic (hypo)manic symptoms. Self-oriented perfectionism was not a significant mediator. For anxiety symptoms the true indirect effects was estimated to lie between 0.06 and 0.33. For stress symptoms the true indirect effects were estimate to lie between 0.06 and 0.31.

*Mediation analyses for probands*

Supplementary Table 2 details the results of the mediation models for the probands in the sample. The same pattern of results was observed. In the two mediation models where ‘chronic depressive symptoms’ was the dependent variable, only self-oriented perfectionism mediated the relationship between both anxiety and stress symptoms and chronic depressive symptoms. Socially-prescribed perfectionism was not a significant mediator. For anxiety symptoms the true indirect effects was estimated to lie between 0.04 and 0.21. For stress symptoms the true indirect effects were estimated to lie between 0.03 and 0.21.

In the two mediation models where ‘chronic (hypo)manic symptoms’ was the dependent variable, only socially-prescribed perfectionism mediated the relationship between both anxiety and stress symptoms and chronic (hypo)manic symptoms. Self-oriented perfectionism was not a significant mediator. For anxiety symptoms the true indirect effects was estimated to lie between 0.07 and 0.45. For stress symptoms the true indirect effects were estimate to lie between 0.07 and 0.5.

| Supplementary Table 1. Unaffected relatives (n=167): Summary of mediation results for chronic depressive and hypomanic symptoms | | | | | |
| --- | --- | --- | --- | --- | --- |
| with self-oriented perfectionism and socially-prescribed perfectionism as mediators controlling for age, gender and | | | | | |
| opposing mood state. |  |  |  |  |  |
|  |  |  |  |  |  |
|  |  |  |  |  |  |
| IV | Effect of IV on DV | Mediators | Effect of IV on M | Effect of M on DV | Indirect effect |
|  |  |  |  |  |  |
| **Chronic Depressive Symptoms** |  |  |  |  |  |
| Anxiety | 0.53** | SOP | 0.78*** | 0.13* | 0.10^a^ |
|  |  | SPP | 0.60** | -0.06 | -0.04 |
|  |  |  |  |  |  |
| Stress | 0.56*** | SOP | 0.76*** | 0.13* | 0.10^a^ |
|  |  | SPP | 0.53*** | -0.04 | 0.01 |
|  |  |  |  |  |  |
| **Chronic (Hypo)manic Symptoms** |  |  |  |  |  |
| Anxiety | 1.07** | SOP | 0.78*** | -0.09 | -0.07 |
|  |  | SPP | 0.60** | 0.26* | 0.16^a^ |
|  |  |  |  |  |  |
| Stress | 1.21*** | SOP | 0.76*** | -0.10 | -0.07 |
|  |  | SPP | 0.53*** | 0.29** | 0.16^a^ |
|  |  |  |  |  |  |
| *Note.* IV, independent variable; M, mediating variable; DV, dependent variable; SOP, Self-Oriented Perfectionism; | | | | | |
| SPP, Socially-Prescribed Perfectionism. | | | | | |
| ^#^*P*<0.10; **P*<0.05; ***P*<0.01; ***P<0.001; ^a^ significant point estimate P<0.05 | | | | | |
|  |  |  |  |  |  |
|  |  |  |  |  |  |
| Supplementary Table 2. **Probands (n=102)**: Summary of mediation results for chronic depressive and hypomanic symptoms with | | | | | |
| self-oriented perfectionism and socially-prescribed perfectionism as mediators controlling for age, gender and | | | | | |
| opposing mood state. |  |  |  |  |  |
|  |  |  |  |  |  |
|  |  |  |  |  |  |
| IV | Effect of IV on DV | Mediators | Effect of IV on M | Effect of M on DV | Indirect effect |
|  |  |  |  |  |  |
| **Chronic Depressive Symptoms** |  |  |  |  |  |
| Anxiety | 0.56*** | SOP | 0.60*** | 0.18* | 0.11^a^ |
|  |  | SPP | 0.56*** | 0.03 | 0.02 |
|  |  |  |  |  |  |
| Stress | 0.58*** | SOP | 0.71*** | 0.16* | 0.11^a^ |
|  |  | SPP | 0.66*** | 0.02 | 0.01 |
|  |  |  |  |  |  |
| **Chronic (Hypo)manic Symptoms** |  |  |  |  |  |
| Anxiety | 0.61^#^ | SOP | 0.60*** | 0.06 | 0.04 |
|  |  | SPP | 0.56*** | 0.40* | 0.22^a^ |
|  |  |  |  |  |  |
| Stress | 0.78^#^ | SOP | 0.71*** | 0.03 | 0.02 |
|  |  | SPP | 0.66*** | 0.38* | 0.25^a^ |
|  |  |  |  |  |  |
| *Note.* IV, independent variable; M, mediating variable; DV, dependent variable; SOP, Self-Oriented Perfectionism; | | | | | |
| SPP, Socially-Prescribed Perfectionism. | | | | | |
| ^#^*P*<0.10; **P*<0.05; ***P*<0.01; ***P<0.001; ^a^ significant point estimate P<0.05 | | | | | |

References

1. Preacher KJ, Hayes AF. Asymptotic and resampling strategies for assessing and comparing indirect effects in multiple mediator models. Behav Res Methods. 2008;40(3):879-91.

2. Preacher KJ, Hayes AF. SPSS and SAS procedures for estimating indirect effects in simple mediation models. Behav Res Methods Instrum Comput. 2004;36(4):717-31.
